# Deregulation of Rho GTPases in cancer

**DOI:** 10.1080/21541248.2016.1173767

**Published:** 2016-04-22

**Authors:** Andrew P. Porter, Alexandra Papaioannou, Angeliki Malliri

**Affiliations:** a Cell Signaling Group, Cancer Research UK Manchester Institute, The University of Manchester, Manchester, UK; b “Cellular and Genetic Etiology, Diagnosis and Treatment of Human Disease” Graduate Program, Medical School, University of Crete, Heraklion, Greece

**Keywords:** cancer, GAPs, GDI, GEFs, Rho GTPases mutations, tumorigenesis

## Abstract

*In vitro* and *in vivo* studies and evidence from human tumors have long implicated Rho GTPase signaling in the formation and dissemination of a range of cancers. Recently next generation sequencing has identified direct mutations of Rho GTPases in human cancers. Moreover, the effects of ablating genes encoding Rho GTPases and their regulators in mouse models, or through pharmacological inhibition, strongly suggests that targeting Rho GTPase signaling could constitute an effective treatment. In this review we will explore the various ways in which Rho signaling can be deregulated in human cancers.

## Introduction

Rho GTPases bind to a wide range of effector proteins and play central roles in the regulation of the actin and microtubule cytoskeletons and gene transcription.^1^ Through these effects, Rho family proteins influence many normal cellular functions such as adhesion, polarity, motility and invasion, as well as cell cycle progression and survival.^2,3^ Rho, Rac and Cdc42 were initially characterized as regulators of the actin cytoskeleton^1^ with a typical pattern being Rho activation leading to the formation of contractile actin, Rac activation controlling peripheral actin structures such as lamellipodia and membrane ruffles, and Cdc42 actin structures such as filopodia.^1^ However, it has long been clear that these proteins have roles far beyond direct regulation of the actin cytoskeleton. For instance, Cdc42 is a master regulator of polarity in organisms from yeast to mammals, while Rac regulates phagocytosis in the immune system, including production of reactive oxygen species.^1,4^ They are involved in many essential physiological processes including embryonic development, neuronal differentiation and neurite formation and maintenance of stem cells in the bone marrow, skin and intestine.^2,3,^^5^ Conversely, deregulation of Rho GTPases is linked to many of the “hallmarks of cancer,” including oncogenic transformation, cell survival and tumor metabolism as well as metastasis (reviewed in ref. 2). While some consequences of deregulated Rho family signaling can be considered pro-tumorigenic, a number of cellular processes stimulated by Rho family proteins—such as the role of Rac1 in apoptosis and maintenance of apicobasal polarity—can be considered to antagonize tumor formation and progression.^6^ The anti-tumorigenic effects of Rho family proteins must be sufficiently differentiated from those pro-oncogenic functions to avoid undermining the therapeutic benefits to be achieved by pharmacologically antagonizing Rho GTPases.

## The Rho GTPase cycle

**Figure 1.**
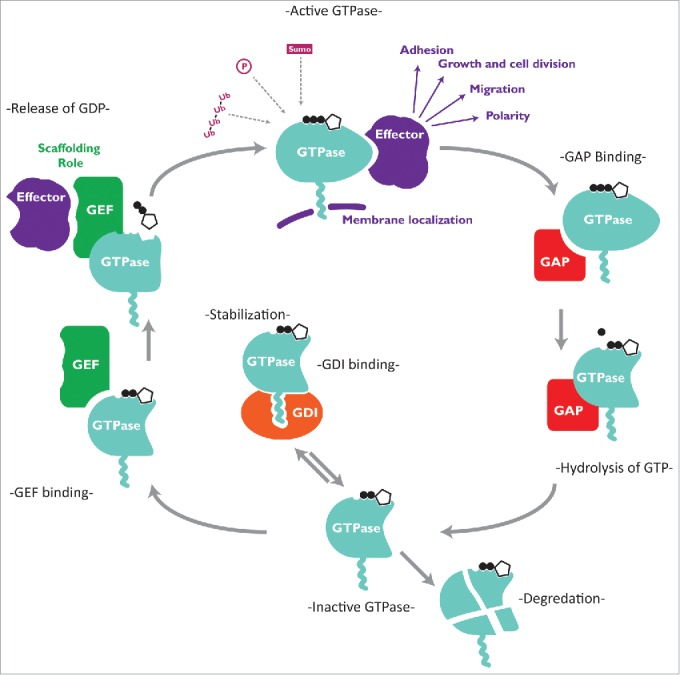
The Rho GTPase cycle GTPase regulation occurs in a number of distinct stages. Guanine nucleotide exchange factors (GEFs) are able to bind to inactive GTPases, displacing the bound GDP, which is then replaced by GTP from the cytoplasm. In their active form Rho GTPases bind to a wide variety of effectors, mediating a large number of cellular processes, including migration, cell-cell adhesion, transcription and proliferation. GEFs also may act to direct signaling by scaffolding particular effectors. To end signaling, GTPase activating proteins (GAPs) bind to the GTPase and enhance their weak intrinsic GTPase activity. Bound GTP is converted to GDP, changing the conformation of the GTPase and rendering it unable to bind effector proteins. Inactive GTPases are mainly found in the cytoplasm, where they can be degraded, or stabilised by binding to Rho GDIs, which act as molecular chaperones and prevent activation by sequestering the GTPases away from GEFs.

Rho GTPases are molecular switches which cycle between an inactive GDP-bound form and an active GTP-bound form (see Fig. 1). The GTPase cycle is largely regulated by guanine nucleotide exchange factors (GEFs) and GTPase-activating proteins (GAPs). ^7^ GEFs displace the GDP bound in the active site allowing GTP binding. GTP binding alters the conformation of the GTPase, allowing it to interact with downstream effector molecules (Fig. 1).^7^ GEFs have also been thought to contribute to signaling specificity through scaffolding upstream and downstream interactors;^8^ this was recently demonstrated with the GEFs Tiam1 and P-Rex1 driving different behaviors via the same small GTPase, Rac1.^9^

Conversely, GAPs activate the weak intrinsic GTPase activity of Rho proteins leading to the hydrolysis of bound GTP, switching the GTPase to an inactive conformation (Fig. 1).^7^ The abundance of GEFs (at least 80) and GAPs (over 70) indicates the importance of tightly controlling Rho GTPase signaling.

Guanine nucleotide dissociation inhibitors (GDIs) are a third class of regulators of Rho proteins. They sequester inactive GTPases in the cytoplasm by masking their C-terminal lipid moieties that mediate plasma membrane localization, which can inhibit their activation^7^ (Fig. 1). They can also protect GTPases from degradation^10^ and also have more subtle effects, such as directing activation of Rho GTPases to specific membrane compartments.^11^ Rho GTPases are also known to be modulated by a host of post-translational modifications, including phosphorylation, ubiquitylation, SUMOylation, ADP-ribosylation, glycosylation, adenylation, and transglutamination/deamidation. Given the wide variety of these modifications, detailed analysis is outside the scope of this review; for more details see refs. 12-14.

**Figure 2.**
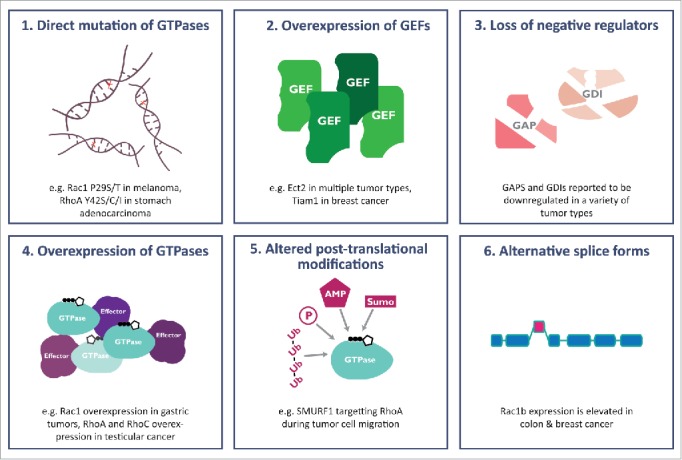
Rho GTPase signaling can be deregulated in cancer by a wide range of mechanisms. (1) Evidence is emerging of many direct mutations of GTPases, such as the Rac1 P29S mutation which is a novel driver in melanoma. (2) GEFs are found overexpressed in many different cancer types, consistent with aberrant Rho GTPase signaling driving transformation and oncogenic progression. (3) Negative regulators of Rho GTPases, such as Rho GAPs and Rho GDIs, have been shown to be tumour suppressors, and lost in human cancers. (4) GTPases are often found to be overexpressed in human cancers, where they drive a variety of oncogenic processes. (5) Post-translational modifications of GTPases, such as changes in ubiquitylation or sumoylation, can alter their signaling. (6) The Rac1b splice form of Rac1 is found in multiple cancers including breast, colon and lung.

At the simplest conceptual level, anything which increases the abundance of the active form should increase signaling, while anything decreasing the abundance of the active state, or actively stabilizing the inactive state, should decrease signaling. Disruption of this balance—by direct activation of Rho GTPases or indirectly through changes in regulators as described above—is increasingly being linked to oncogenesis (see Fig. 2). In this review we will focus on the variety of ways in which Rho signaling has been shown to be disrupted in cancer: alterations in protein levels of the GTPases, disruption to regulators of GTPases, changes in post-translational modifications of GTPases and finally we review the emerging literature on direct mutation of GTPases.

## Copy number alterations and misexpression of Rho GTPases in cancer

Before the finding of direct mutations of Rho GTPases, the main way they were thought to be misregulated in cancer was through changes in expression levels (see Fig. 2). Increased expression of Rho proteins is often associated with tumor formation, growth and progression, an indication of a positive contribution of increased Rho GTPase activity to tumorigenesis.^2^ The interesting exception is RhoB which, as discussed below, appears to more commonly play a tumor suppressor role, and is accordingly found at reduced levels in tumor samples.

Rac1 has been found to be overexpressed in testicular, breast and prostate cancer, as well as gastric and lung cancers.^15-19^ In recent studies, its overexpression in gastric cancer was correlated with the aggressiveness of the tumors, greater invasion and lymph node metastasis, as well as poor patient survival.^19^ Rac1 is also overexpressed in acute myeloid leukemia cells, where it enhances migration and cell growth, and is linked to chemoresistance.^20^

Animal experiments support a requirement for Rac1 in tumor formation and growth in many different tumor models. Mice with Rac1 deletion specifically from keratinocytes are resistant to developing Ras-induced skin cancer^21^ while those with a Rac1 deletion in pancreatic progenitor cells are protected from development of pancreatic ductal adenocarcinoma (PDAC).^22^ Rac1 is also required for K-Ras-induced lung tumors in mice,^23^ and cooperates with APC loss in a mouse model of colorectal cancer, driving a stem-cell like signature in the developing cancer cells.^24^ Another recent study showed that Rac1 affects stem cell behavior to drive oncogenic progression, by reducing the differentiation of tumor cells.^25^

A splice variant of Rac1, Rac1b, was found at elevated levels in colon and breast tumors.^26^ Rac1b includes an additional 14 amino acids compared with wild-type Rac1 and it is mainly found in its active GTP-bound state. Rac1b has reduced affinity for GDIs, meaning it is not sequestered in the cytoplasm, which could explain its increased activity and ability for cellular transformation.^27^ Rac1b alone is insufficient to drive tumor formation in a non-small cell lung cancer mouse model, but it enhances the activity of K-ras mutations.^28^ It is highly expressed in stages 1 and 2 of human lung adenocarcinoma, making it a candidate target for preventing progression to more aggressive stages.^29^ Rac1b is also overexpressed in papillary thyroid carcinoma (PTC), where it is associated with the BRAF V600E mutations and subsequently with poor clinical outcomes.^30^

Rac2, which shares a high degree of sequence conservation with Rac1, is restricted to expression in the haematapoetic cell lineage. Although no aberrations of Rac2 have been directly linked to oncogenesis, Rac2 is emerging as a therapeutic target, as abrogation of Rac2 signaling slows the growth of AML and CML tumors (reviewed in ref. 31). Rac3 activity was found to be increased in highly proliferative breast cancer cell lines, although this does not correspond to increases at the protein level^32^ suggesting other mechanisms of activation.

RhoA and RhoC have been found overexpressed in a wide range of tumors, particularly those with epithelial origins,^2^ and in some instances have been linked to oncogenic progression, such as in testicular cancer^15^ and to poor prognosis, such as in esophageal squamous cell carcinoma.^33^ In contrast to overexpression, loss or reduced expression of RhoB was observed in lung cancer and head and neck squamous cell carcinoma^34,35^ suggesting that loss of function of RhoB can contribute to oncogenic progression. However, in a contradictory finding, RhoB is found overexpressed in breast cancer,^16^ which suggests possible cell- or cancer-type specific roles for this GTPase which may result from differential expression of downstream effectors and/or upstream scaffolding proteins, or the balance between other Rho GTPases.

Analysis of gene expression data from the SAGE database reveals changes in Cdc42 levels in cancer tissue, both increased and decreased, compared to normal tissue.^36^ Cdc42 is overexpressed in testicular and breast cancer,^15,16,^^37^ in non-small cell lung cancer,^38^ and in colorectal adenocarcinoma and cutaneous melanoma.^39,40^ Finally a less well-studied GTPase, Rnd3/RhoE is downregulated in HCC (hepatocellular carcinoma) and its downregulation is correlated with poor prognosis and tumor progression,^41,42^ while it is upregulated in gastric cancer cells under hypoxic conditions promoting EMT,^43^ again highlighting the signaling complexity of these GTPases and their downstream targets.

The evidence for altered expression of the above mentioned GTPases is indicative of a role in tumor initiation and/or progression. It should also be considered that lack of data for some of the lesser-studied members of the Rho GTPase family may in part be due to fewer reagents being available with which to look for alternations in these proteins. More unbiased screening, and particularly genome-level sequencing for activating mutations (see below), may help to reverse some of this historical bias.

## Indirect regulation of Rho GTPases in cancer

### Modulation of Rho family regulators

An alternative mechanism by which many tumors upregulate Rho GTPase signaling is by changing the levels or activities of GTPase regulators, including GEFs, GAPs and Rho GDIs (Fig. 2).^44,45^ While the general trend is toward overexpression of GEFs, and reduced expression of GAPs and GDIs (indicative of a positive contribution of Rho GTPase signaling to tumorigenesis) the detailed picture emerging is of much more complex regulation, seemingly dependent on tumor type and level of progression.

Τhe GEFs Ect2, MyoGEF, P-Rex1, Tiam1, LARG, Dock180, Vav1, Vav2, Vav3 and β-PIX are overexpressed in a variety of human tumors.^46^ Ect2, which has activity for multiple members of the Rho GTPase family including RhoA, Rac1 and Cdc42, has been recognized as an oncogene in human cancer since 2010, being aberrantly overexpressed and mislocalised in various types of tumors.^47^ Activation of MyoGEF – a RhoA and RhoC GEF - regulates the invasion of breast cancer cells.^48^ Overexpression of the Rac1 GEF P-Rex1 promotes metastasis of prostate cancer^49^ and mutations have been identified in PREX2 (a Rac GEF) in melanoma.^50^ Tiam1, another Rac1 GEF, was initially identified as being important for invasion in T-cell lymphoma.^51^ Tiam1 displays high levels of expression in breast cancer where it is associated with grade and metastatic potential^52^ and is a marker for poor prognosis.^53^ Overexpression of Tiam1 has also been observed in prostate cancer.^17^ Furthermore, overexpression of Tiam1 in lung adenocarcinomas as well as in squamous-cell carcinoma of the head and neck (SCCHN) is associated with disease progression and poor patient survival.^54^ In lung cancer, levels of Tiam1 inversely correlate with expression of the E3 ubiquitin ligase HUWE1, which degrades Tiam1 specifically from cell-cell adhesions, indicating that localized regulation of GEF abundance may play a role in cancer.^55^ The Tiam1 ortholog STEF/Tiam2 was found to promote proliferation and invasion in liver cancer when overexpressed, and is therefore implicated in the pathogenesis of HCC.^56^ β-PIX has also been found overexpressed in many breast cancers.^57^ The haematopoietic specific GEF Vav1 is ectopically expressed in pancreatic adenocarcinoma with a positive correlation to reduced patient survival^58^ and its presence in a subset of neuroblastoma tumors indicates its involvement in the tumorigenesis process.^59^ Moreover, high levels of expression of Vav1 are a marker for poor prognosis in breast cancer.^53^ The Vav1 orthologues Vav2 and Vav3 have also been shown to be deregulated in human tumors. Vav3 is overexpressed in gastric cancer^60^ as well as in prostate cancer where a novel nuclear function was found to be responsible for its role in malignant progression.^61^ Moreover, both Vav2 and Vav3 regulate a lung-metastasis specific transcriptome that leads to breast cancer progression.^62^ Finally, the bipartite Rac1 GEF composed of Dock180 and ELMO1 is overexpressed in malignant gliomas, where it contributes to invasion,^63^ whereas LARG (leukemia-associated Rho GEF) is found fused with the MLL locus in acute myeloid leukemia (AML)^64^ leading to aberrant expression. While not exhaustive, this list is highly indicative of an oncogenic function for upregulated Rho GTPase signaling.

This data from human tumors is supported by evidence from transgenic mouse models highlighting the importance of a number of GEFs in oncogenic progression. Tiam1 has been shown to be important for Ras-mediated skin^65^ and intestinal tumorigenesis.^66^ Interestingly, Tiam1 deficient mice develop fewer tumors, but those which do grow are more invasive, suggesting both positive and negative roles for Tiam1 in oncogenesis. Loss of P-Rex1 leads to a reduction in the invasive potential of melanoma cells in a mouse model of the disease, consistent with work *in vitro* showing that P-Rex1 can regulate invasion.^67^ P-Rex2 is also frequently mutated in melanoma, and a truncating mutant, E824*, has recently been shown to cooperates with NRAS to accelerate melanoma development in a mouse model.^68^

Mice deficient for the Rac1/Cdc42 GEFs Asef1 and Asef2, which are downstream of APC and are overexpressed in colorectal tumors, show reduced spontaneous formation of intestinal adenomas.^69^ Mice transplanted with leukemic B-cell progenitors expressing the p190-BCR-ABL transgene develop tumors at high frequency; however if these cells are deficient for Vav3 then tumor formation is significantly decreased, and survival time increased.^70^ Both Vav2 and Vav3 are required for initiation and promotion of skin tumorigenesis.^71^

The GAP DLC1 (deleted in liver cancer) is a tumor suppressor frequently downregulated in many cancer types either by deletion or epigenetic silencing. Loss of DLC1 leads to an activation of RhoA, and cooperates with oncogenic Myc in a mouse model of liver cancer.^72^ DLC2 was also found downregulated in hepatocellular carcinoma,^73^ and more recently was shown to be required to regulate Cdc42 activity for faithful chromosome segregation during mitosis.^74^ P190RhoGAP is another RhoGAP thought to act as a tumor suppressor; it is frequently deleted in gliomas, and its overexpression is able to suppress tumor formation in a mouse model of the disease.^75^ However not all GAPs are found downregulated in human tumors; ARHGAP8 is found overexpressed in colon cancer.^76^

The picture for Rho GDIs is relatively complex, possibly due to their ability to target multiple Rho GTPases and their roles in regulating Rho GTPase activity, stability and trafficking.^11^ For instance, Rho GDI1 is found downregulated in some breast cancer studies,^77^ but overexpressed in others.^78^ Downregulation of Rho GDI2 in bladder cancer is associated with decreased patient survival^79^ whereas overexpression in pancreatic cancer is associated with invasion. ^80^

## Post-translational modifications

As discussed earlier, Rho GTPases are regulated by a whole host of post-translational modifications, many of which are now being linked to inappropriate Rho GTPase function in human cancers and a few of which we will discuss here as illustrative examples. Ubiquitylation of Rac1, RhoA and Cdc42 can be deregulated in cancer cell lines, a fact that could indicate a link between Rho GTPase protein ubiquitylation and cancer.^14^ For instance, the E3 ligase SMURF1 targets RhoA for degradation at the leading edge of migrating cells, affecting tumor cell migration.^81^ PIAS3 SUMOylates Rac1 stabilizing the active form of the protein following HGF stimulation and therefore promoting cell migration and invasion, suggesting a possible role in cancer progression.^13^ Conversely, Rac1 can be ubiquitylated by the E3 ligase HACE1, resulting in its proteasomal degradation, reducing Rac1 mediated migration.^82^ Ubiquitylation of RhoA has also been reported to be impaired following FBXL19 downregulation in lung cancer epithelial cells.^83^ FBXL19 ligase also ubiquitylates Rac1 and Rac3, with degradation impairing esophageal cancer cell EMT.^84^ Finally, phosphorylation of Rho GTPases has also been shown to regulate their transforming ability; for instance phosphorylation of Cdc42 by the Src tyrosine kinase modulates its interaction with Rho GDI which is necessary for cellular transformation.^85^ These examples from the literature demonstrate some of the great diversity of mechanisms by which cancer cells can indirectly disrupt upstream signals which lead to Rho GTPase activation.

## Direct mutations of GTPases in human cancers

Early studies had identified mutations in RhoH such as the rearrangement of RhoH/TTF gene and the mutation of the 5′-UTR of RhoH gene in some haematopoietic malignancies.^86,87^ However, mutations within Rho GTPases, except for RhoH, were believed to be rare in cancer until recently. This led to the speculation that Rho GTPases were not direct drivers of oncogenic progression, but merely downstream players in a disease more directly modulated by upstream signaling pathways. With the development of faster and cheaper deep sequencing technology this idea has been challenged, as Rho GTPases have now been found mutated in a wide variety of cancer types (see Table 1).^88^ In particular, the discovery of a recurrent Rac1 mutation in melanoma has significantly altered the perception of the role of Rho GTPases as drivers of oncogenic progression. For this review, we gathered data on published mutations in the Rho GTPases Rac1, Rac2, Rac3, Cdc42, RhoA, RhoB, RhoC, RhoH and RhoT1 using the cBio portal (http://www.cbioportal.org/), a database that collects cancer genomics data sets from tumor samples across cancer studies,^89,90^ and IntOGen (https://www.intogen.org/search), which assesses mutational data across multiple tumor types to identify potential driver mutations.^91^ Both databases are user-friendly, regularly updated, and include additional information such as expression levels, amplifications and deletions (see Table 1). While any table of this kind becomes quickly outdated, it nonetheless serves to highlight the remarkable impact of sequencing technology on the discovery of mutations in human cancers in recent years, as well as the range of cancer types harboring mutations in Rho GTPases. The following section will focus on the emerging literature around these newly-identified mutations and other identified deregulations of Rho GTPases in human cancers.

**Table 1.** A search was conducted using the cBioportal and IntoGen databases for mutations in the Rho GTPases Rac1, Rac2, Rac3, Cdc42, RhoA, RhoB, RhoC, RhoH and RhoT1 which occurred in samples from human patients, and which have been published in the literature. We did not include mutations from cancer cell lines, or provisional data uploaded on the sites.

Rho GTPase	Acute Myeloid Leukemia	Bladder Urothelial Carcinoma	Breast Invasive Carcinoma	Clear Cell Renal Cell Carcinoma	Colorectal Adenocarcinoma	Cutaneous squamous cell carcinoma	Esophageal Adenocarcinoma/Esophageal Squamous Cell Carcinoma/Nasopharyngeal Carcinoma	Pediatric Ewing Sarcoma	Glioma and Glioblastoma	Head and Neck Squamous Cell Carcinoma	Liver Hepatocellular Carcinoma	Lung Adenocarcinoma	Lung Squamous Cell Carcinoma and Small Cell Lung Cancer	Multiple Myeloma	Malignant Peripheral Nerve Sheath Tumors	Non-renal clear cell carcinoma	Ovarian Serous Cystadeno-carcinoma	Papillary Thyroid Carcinoma	Prostate adenocarcinoma	Skin Cutaneous Melanoma	Stomach Adenocarcinoma	Uterine Corpus Endometrial Carcinoma
Cdc42	S30L	F110L		S185C	R68*					A41T		Q116E	E95Q					D63V		G12V (2)	L70P	E62D, E127D
					R186H							D122N									K166E	K150N
Rac1		G30E	P69S	Y40S	R68H			A159V		G15S		A159V	V46G	N92K				N111I	Q61R	**P29S (12)**	P34H	**P29S**
		L53V								C18F, C18Y			R102L							D65N		
										**P29S, P29T**												
										N39S												
										K116N, K116R												
										G142S, A159V (2)												
Rac2				X12_splice	V36A		V168M		R102W	I21M		C189S	K147M						V93I	A27V, P29L (2)	G15D	C18R
										D124E									R187H	E62K, R102Q	V168M	D65N
																				R174W	P136H	F82L
Rac3				A95V						P34L					Y23H				S89Pfs*64	X76_splice (2)	V44M, E100D	
										S158*									A88T	A135T	R102W, P185L	
		**R5W (3)**			**R5Q**		**R5L**		I80T	**E40Q (5)**		E40*					G17V		D59G	P75S	**R5W (2)**	V24F
		G17A (2)			T37I		**R5W**			**Y42I**		D59N					D76N			S85F	G17E, L22R	A148T
		L22P, S26R			A61V		Y34C														Y34C, F39C	T175M
		**E40Q (2)**			L69P		G184E														**E40K**	
RhoA		Q63K, P75R			S85Ffs*6																N41K	
		F106L, E125Q			F154C																**Y42C (7),Y42S (2)**	
		E142K, D146H																			L57V (4), D59Y	
		A161V (2)																			T60K, A61D	
		R168T, E172K																			G62E (2), G62R	
		E172K, S188Ifs*30																			Y74D	
		G14S, E47K (2)	D13Y (2)	S26T (2)	F30L				G12D	E165K	A15S	D13Y	A2S				V127G			L81F	V9L	S88N
		Y66_R68del	D13Y	Y34C								P108R									R133S	
		P75S (2)	W58C																			
RhoB		P75T, P75L	D59N																			
		K135Q, E158K	E125K																			
		K162N, E172K (2)	E169K																			
		Q180P																				
			Y42C		D120N			D146E		E125Q		R145W	S73A			L22H	D59E		E64K		G178D	R68Q
RhoC			E142K		R150W								K162N									S73Lfs*5
																						R182S
		E135K			D58G, R69Q	S129F	S155G		V167I			C7*, F65L	A32T, E39K				R23H			P35S, G49S	R23C	S53T
RhoH					R177Q							R121L, R127M	Y83C, S84Y							E101K, R168Q		
		R104K, Q213E	E39Q (2)		E12K, E353*		X110_splice		E505K		R104K	C377F	S156L, R234G				D106H		N422D	P30L, P43S	R50Sfs*15	K230N, L307V
RhoTI		E300K, S479L	D91N (2), D91N		I407Dfs*16		P326L						R261S, D317Y				A305V			R191C (2)	Y82H (2)	V418M, R450C
		E505Q	K230N, E285G (2)		K412Nfs*12								E484A, T543A				V418L			P220L, K405*	R263T	A458V (2)
					N428Tfs*16																I407Dfs*16	
					K469I																	

*Notes.* * = Stop codon; fs = Frame shift; Mutations in bold = Hotspot mutation; Solid boxes = Identified as a driver mutation by IntOGen algorithms. Numbers in brackets indicate number of times a mutation identified across data sets.

## Rac1 mutations

One early study aiming to detect Rac1 mutations in human brain tumors identified deletions, frame shift and point mutations in 12 out of 45 samples from human patients with brain tumors,^92^ suggestive of a role for Rac1 in brain tumor development. Now, next generation sequencing has identified a number of cancer-associated mutations along the length of the Rac1 protein, with Rac1 being identified as a driver mutation in head and neck squamous cell carcinoma and cutaneous melanoma (see Table). Among these, P29 is a hot-spot for Rac1 mutation. It was first identified by 2 groups in 2012.^93,94^ Whole-exome sequencing was performed in melanoma samples and 5% of them were found to harbour missense mutations in the Rac1 gene, making Rac1 the third most highly mutated gene in melanoma (after BRaf and NRas).^93^ The functional effect of the P29S recurrent mutation is to induce a ‘fast cycling’ form of Rac1, as opposed to the more common gain-of-function mutations used in a laboratory setting which are modeled on activating Ras mutations found at high frequency in human cancers. These mutations, found at G12 and Q61, block GTPase activity and so trap the GTPase in its active, GTP-bound form. In contrast, the P29 residue lies in a hydrophobic pocket in the switch I region of the Rac1 GDP-bound form, and the substitution of the proline residue for a serine enhances the exchange of GDP for GTP,^95^ while still maintaining the ability to hydrolyse GTP back to GDP. Overall this enhances the interaction of Rac1 with effectors, such as the Pak family of kinases. P29S is therefore considered a gain-of-function mutation that likely promotes oncogenic events during melanoma through mechanisms thought to include altered cell proliferation, adhesion, migration and invasion.^93^ Expression of the mutant form of Rac1 in melanocytes leads to enhanced cell proliferation and migration,^94^ and the Rac1 P29S mutant form is able to transform mouse fibroblasts and immortalised breast epithelial MCF10A cells.^96^ Subsequently 2 other fast-cycling mutants of Rac1 have been identified, N92I and C157Y.^96^ The ability to cycle from the off-state to the on-state may render these fast-cycling mutants more efficient at driving transformation than the constitutively active mutants, possibly because they more closely mimic normal signaling by being able to associate and dissociate from effectors, or potentially by still associating with GEFs acting also as scaffolding proteins. Rac1 N92I was able to efficiently transform mouse fibroblasts and MCF10A cells, whereas the C157Y mutation was less effective.^96^ Interestingly, Rac1 P29S (which has also been found as a somatic mutation in a breast cancer cell line) transformed MCF10A cells more efficiently than fibroblasts, whereas the opposite was true for the Rac1 N92I mutation (known as a somatic mutation in a fibrosarcoma cell line),^96^ suggesting that there are further subtleties to the effects of these different activating mutations still to be uncovered.

A serious clinical problem in the treatment of melanoma is the swift development of resistance to the front line treatments of RAF and MEK inhibition. A 2014 study revealed that Rac1 P29S expression in melanoma cell lines and in mouse tumor models conferred resistance to RAF and MEK inhibitors^97^ with overexpression of Rac1 P29S decreasing apoptosis after RAF and MEK inhibitor treatment. A further clinical study suggested the potential of Rac1 P29S as a predictive biomarker for resistance to therapy in melanoma patients under treatment with these inhibitors.^98^ Further histological and clinical evidence showed that this hot spot mutation may be responsible for the early metastatic progression of BRAF mutant and BRAF wild-type melanoma.^99^ A more recent biological insight into the P29S mutation showed increased expression of PD-L1 in Rac1 P29S melanoma patients compared to Rac1 wild type or other Rac1 mutants.^100^ PD-L1 is a suppressor of the immune system thus its upregulation may allow cancers to evade the host immune system and therefore oncogenic Rac1 P29S may promote the reduction of anti-tumor immune response. As PD-L1 is a candidate biomarker for increased benefit from treatment with anti-PD1 or anti-PD-L1 antibodies, this finding could also have implications in the clinic.

## Rac2 and Rac3 mutations

In the 2012 study conducted by Hodis et al., a homolog to the Rac1 P29 residue was found to be mutated in Rac2, substituting Proline (P) with Leucine (L) (P29L mutation).^93^ Two Rac2 mutations - P29L and P29Q – similar to the P29S mutation, were confirmed as transforming mutations of Rac2.^96^ Additional mutations have since been identified in Rac2 (see Table 1) and among these the R102Q was found as a hot spot mutation. Mutations in the Rac3 gene have been identified from a range of cancers, including melanoma, stomach and prostate, but none has yet been studied functionally. Rac2-KO and Rac3-KO mice showed slightly increased survival in a CML and ALL background, respectively,^101,102^ suggesting a possible oncogenic role for these genes; further experimentation will be required to determine the functional significance of these cancer-associated mutations.

## RhoA mutations

As with Rac1, no mutations in RhoA had been detected in human cancers until very recently. RhoA mutations were identified by several groups in 2014,^103^ with an IntOGen search indicating a driver role for RhoA in stomach adenocarcinoma (see Table 1), as well as a general pan-cancer driver role. RhoA mutations have been identified in 25% of diffuse-type gastric carcinoma cases studied.^104^ Recurrent mutations were R5Q, G17E and Y42C. Expression of both RhoA G17E and Y42C were able to rescue growth defects of SW948 colon cancer cells grown in 3D culture following knockdown of endogenous RhoA in contrast to re-expression of wild-type RhoA which was unable to rescue.^104^ Several groups have found frequent RhoA mutations, specifically the G17V mutation, in angioimmunoblastic T cell lymphoma and peripheral T cell lymphomas.^105-107^ Interestingly this mutation appears to act similarly to well-characterized dominant negative mutations of RhoA, rather than as an activating mutation. Expression of this mutant form of RhoA increases proliferation in Jurkat cells, an effect also observed with expression of dominant negative RhoA. This fits well with work showing that inactivation of RhoA promotes tumor formation in colorectal cancer models.^108^ Silencing of RhoA in colon cancer cell lines promoted proliferation, largely through activation of the Wnt/β-catenin pathway and subsequent upregulation of Myc signaling, and this led to increased metastasis. In a mouse model of colorectal cancer, metastatic sites were found to have lower RhoA signaling than the primary tumors, and this held for samples from human tumors as well.^108^ Another example of inactivating RhoA mutations are those found recurrently in Burkitt Lymphoma, the most common type of childhood B-cell lymphoma. Translocations of the MYC locus leading to deregulated Myc signaling are necessary but not sufficient to drive disease progression, and both whole genome studies^109^ and exome sequencing^110^ identified RhoA mutations as additional drivers of the disease. 8.5% of cases had RhoA mutations, and molecular modeling of these mutations suggested that they would reduce RhoA activity, or reduce binding to RhoA effectors.^110^

Another study conducted with gastric adenocarcinoma samples^103^ added a number of additional mutations including Y34C, F39C, E40K, N41K, Y42S/C/I, L57V, D59Y, T60K, A61D and G62R/E (and see Table 1). These were accumulated in regions that participate in the interaction of RhoA protein with effector molecules; for instance mutations at Y42 reduce downstream activation of PKN but not mDia or ROCK1.^111^ This indicates that distinct mutants may have different alterations in effector binding/activation with some of them leading to reduced interaction of RhoA with specific effector proteins. Depending on the target affected, this altered RhoA activity could account for the increased cell spread and the absence of cell cohesion observed in this kind of tumors. These studies suggest either that wild-type RhoA, in the cells of origin for these cancer types, is acting in a tumor suppressive capacity, or that inactivation of RhoA in some way leads to hyperactivation of an oncogenic pathway. C3 toxin-mediated inactivation of RhoA, B and C causes the development of aggressive malignant thymic lymphomas in mice.^112^ Such findings support a tumor suppressor role for these members of the Rho family. It will require further experiments to reconcile data from these mutational studies with earlier work showing that overexpression of RhoA promotes tumorigenesis. This could be due to differences in the expression of downstream effectors in different tissue types, or different requirements for RhoA throughout the life-cycle of a tumor.

## RhoB, RhoC and RhoT1 mutations

RhoB has been found to be mutated in 5% of bladder cancer cases from a sample of 131 high grade tumors not treated with chemotherapy (with more than 200 additional samples still to be sequenced at the time of writing) making it one of 9 genes mutated in this disease.^113^ Our cBio search for published RhoB mutations (see Table 1) indicates that P75S/T/L is a hot spot mutation, though it has not yet been studied functionally. In a model of Ras-driven skin cancer, Liu and colleagues showed that the RhoB-null mice had increased skin tumors compared to the heterozygote mice and that RhoB-deficient MEFs transformed with E1A and Ras showed greater resistance to DNA-damage induced apoptosis,^114^ which suggests that, if functional, these might be inactivating mutations.

Two other family members, RhoC and RhoT1, present a number of published mutations in cancer samples and cell lines, with the S73 residue a hotspot in RhoC, while mutations in RhoT1 include a P30L mutation, which by homology may have similar effects to the Rac1 P29S mutation. Deletion of RhoC from mice has been observed to reduce the frequency and growth of tumors,^115^ which might suggest that activating mutations might promote tumor formation, but further analysis of mutations in these family members is required to determine their functional relevance.

## Cdc42 mutations

The classical activating mutation G12D (equivalent to the G12V activation mutation of Ras) has been found in Cdc42 in melanoma cells in the same study which identified the Rac1 P29S mutation,^93^ although this mutation was present in only a single patient sample, and has not been functionally characterized. Table lists 14 different published mutations in Cdc42, although no function has yet been ascribed to them. However, given the evidence for a role for Cdc42 in cellular transformation,^36^ we conclude that it is highly likely that at least some of these mutations will be functionally active. It is also possible that some of these may be inactivating mutations, as *in vivo* evidence, such as deletion of Cdc42 from hepatocytes which lead to spontaneous tumor formation,^116^ suggests that Cdc42 might also play a role as a tumor suppressor.

## Pharmacological inhibition of Rho GTPases

Given the long-standing *in vitro* and *in vivo* data showing Rho protein involvement in malignant transformation, observed changes in Rho protein expression levels or changes in their regulators and post-translational modifications, and now direct mutation of Rho GTPases, in human cancers, targeting Rho protein signaling is an increasingly attractive target for new cancer therapeutics. Small molecule inhibitors of many Rho proteins are currently being developed and tested.

Two different small molecule inhibitors of Rac are currently in use, utilizing 2 different strategies for inhibition. NSC23766 works by inhibiting the interaction between Rac1 and its GEF Tiam1, reducing the activation of Rac1.^117^ EHT 1864 is a pan-Rac inhibitor which directly targets the Rac GTPase itself, by displacing GTP from the active site.^118^

NSC23766 can halt the proliferation, anchorage-independent growth and invasion of prostate cancer cells.^117^ Rac1 inhibition can additionally reduce growth of non-small-cell lung cancer tumors in a mouse model that present resistance to (EGFR)-tyrosine kinase inhibitors such as gefitinib, making it attractive as a potential combination therapy to help circumvent the resistance mechanisms.^119^ Moreover, Rac1 inhibition impedes the growth, invasion and metastasis of gastric tumors.^19^ However, while both these inhibitors do indeed target Rac activity, they also have significant off-target effects, as demonstrated by assays using wild-type and Rac1-deficient mouse platelets.^120^ This emphasizes the need to develop better versions of these drugs, or find other ways of targeting Rac, and other small GTPases. One approach is to use in silico screening to predict potential binding partners which might block GTPase-GEF interactions.^121^ It is worth noting that this strategy of targeting the interaction between GEFs and GTPases is predicated on the function of the GEFs regulating tumorigenesis via their ability to activate the GTPases. However, this is not always the case. For instance, the activation of the PI3K/Akt pathway by the GEF P-Rex2 does not depend on the GEF activity of the protein.^122^ Also, a recent paper from our lab demonstrates that different GEFs can have differential effects on cell behavior, despite activating the same GTPase to similar levels,^9^ most likely by scaffolding different downstream effectors of the GTPase; therefore it will be important to target the correct GEF-GTPase activity for the specific cancer type.

Given that Rac1 and Cdc42 are highly expressed and active in ovarian cancer,^123^ inhibitors of these 2 GTPases have been tested in immortalized and primary human ovarian cancer cells.^124^ The R enantiomer of ketorolac, (ketorolac is given as an anti-inflammatory drug), can inhibit Rac1 and Cdc42 and was shown to improve patient outcomes in treatment for ovarian cancer.^124^ Another Rac1 and Cdc42 dual-inhibitor, AZA1, identified from a screen of molecules based on modifying the structure of NSC23766, has been used in *in vitro* studies to target prostate cancer cells.^125^ This synthetic compound reduced cancer cell migration and proliferation and succeeded in increasing the survival of xenograft mouse models of prostate cancer by targeting Rac1 and Cdc42 but not RhoA.^125^

An additional 3 inhibitors of Cdc42 have been developed. Secramine has been identified as a small molecule inhibitor that perturbs Cdc42 activity in a RhoGDI1-dependent manner,^126^ although is likely to affect other GTPases in the same manner. ZCL278 is a small molecule inhibitor of Cdc42, designed to block the interaction of Cdc42 with the GEF Intersectin. It is thought to disrupt both GEF interactions and GTP binding^127^ and was shown to inhibit actin-based motility and migration in a metastatic prostate cancer cell line.^127^ Finally, AZA197, another recently developed Cdc42 inhibitor which appears not to inhibit Rac1 activity has shown some efficacy in reducing tumor size in a xenograft model of colon cancer.^128^

Reducing signaling through the Rho pathway is often achieved by targeting the Rho target ROCK.^129^ The ROCK inhibitor Y-27632 retards migration of human prostate cancer cells in mice^130^ and blocks the invasive activity of cultured rat hepatoma cells.^131^ Moreover, inhibiting the Rho/ROCK signaling pathway in NSCLC using the ROCK inhibitor fasudil, when combined with inhibition of the proteasome, effectively reduced the viability of mutant K-Ras cells compared with wild-type cells.^132^

It is likely that further structural modification of these compounds, or further high-throughput compound-screening, will lead to more specific inhibitors, and that as we further our understanding of both normal and abnormal Rho GTPase signaling we will be better placed to deploy them therapeutically.

## Conclusion

In conclusion, Rho GTPase signaling is frequently seen to be modified in human cancers through a variety of mechanisms, and work is continuing to understand the consequences of this aberrant signaling. Understanding the wider landscape of Rho GTPase signaling in a tumor type is likely to be important for making the correct, clinically-relevant interventions. Modifications occur from the level of mutation of the GTPases to under or overexpression of their regulating proteins, which both generates a highly complex signaling network that needs further work to be untangled and also suggests many fertile avenues for therapeutic intervention.

## References

[1] Hall A. Rho family GTPases. Biochem Soc Trans 2012; 40:1378-82; PMID:23176484; http://dx.doi.org/10.1042/BST20120103 23176484

[2] Orgaz JL, Herraiz C, Sanz-Moreno V. Rho GTPases modulate malignant transformation of tumor cells. Small GTPases 2014; 5:e29019; PMID:25036871; http://dx.doi.org/10.4161/sgtp.29019 25036871 PMC4125382

[3] Sadok A, Marshall CJ. Rho GTPases: masters of cell migration. Small GTPases 2014; 5:e29710; PMID:24978113; http://dx.doi.org/10.4161/sgtp.29710 24978113 PMC4107589

[4] Knaus UG, Heyworth PG, Evans T, Curnutte JT, Bokoch GM. Regulation of phagocyte oxygen radical production by the GTP-binding protein Rac 2. Science 1991; 254:1512-5; PMID:1660188; http://dx.doi.org/10.1126/science.1660188 1660188

[5] Jaffe AB, Hall A. Rho GTPases: biochemistry and biology. Annu Rev Cell Dev Biol 2005; 21:247-69; PMID:16212495; http://dx.doi.org/10.1146/annurev.cellbio.21.020604.150721 16212495

[6] Mack NA, Whalley HJ, Castillo-Lluva S, Malliri A. The diverse roles of Rac signaling in tumorigenesis. Cell Cycle 2011; 10:1571-81; PMID:21478669; http://dx.doi.org/10.4161/cc.10.10.15612 21478669 PMC3127158

[7] Cherfils J, Zeghouf M. Regulation of small GTPases by GEFs, GAPs, and GDIs. Physiol Rev 2013; 93:269-309; PMID:23303910; http://dx.doi.org/10.1152/physrev.00003.2012 23303910

[8] Rossman KL, Der CJ, Sondek J. GEF means go: turning on RHO GTPases with guanine nucleotide-exchange factors. Nat Rev Mol Cell Biol 2005; 6:167-80; PMID:15688002; http://dx.doi.org/10.1038/nrm1587 15688002

[9] Marei H, Carpy A, Woroniuk A, Vennin C, White G, Timpson P, Macek B, Malliri A. Differential Rac1 signalling by guanine nucleotide exchange factors implicates FLII in regulating Rac1-driven cell migration. Nat Commun 2016; 7:10664; PMID:26887924; http://dx.doi.org/10.1038/ncomms10664 26887924 PMC4759627

[10] Boulter E, Garcia-Mata R, Guilluy C, Dubash A, Rossi G, Brennwald PJ, Burridge K. Regulation of Rho GTPase crosstalk, degradation and activity by RhoGDI1. Nat Cell Biol 2010; 12:477-83; PMID:20400958; http://dx.doi.org/10.1038/ncb2049 20400958 PMC2866742

[11] Garcia-Mata R, Boulter E, Burridge K. The ‘invisible hand’: regulation of RHO GTPases by RHOGDIs. Nat Rev Mol Cell Biol 2011; 12:493-504; PMID:21779026; http://dx.doi.org/10.1038/nrm3153 21779026 PMC3260518

[12] Nethe M, Hordijk PL. The role of ubiquitylation and degradation in RhoGTPase signalling. J Cell Sci 2010; 123:4011-8; PMID:21084561; http://dx.doi.org/10.1242/jcs.078360 21084561

[13] Castillo-Lluva S, Tatham MH, Jones RC, Jaffray EG, Edmondson RD, Hay RT, Malliri A. SUMOylation of the GTPase Rac1 is required for optimal cell migration. Nat Cell Biol 2010; 12:1078-85; PMID:20935639; http://dx.doi.org/10.1038/ncb2112 20935639 PMC2992316

[14] Visvikis O, Maddugoda MP, Lemichez E. Direct modifications of Rho proteins: deconstructing GTPase regulation. Biol Cell 2010; 102:377-89; PMID:20377524; http://dx.doi.org/10.1042/BC20090151 20377524

[15] Kamai T, Yamanishi T, Shirataki H, Takagi K, Asami H, Ito Y, Yoshida K. Overexpression of RhoA, Rac1, and Cdc42 GTPases is associated with progression in testicular cancer. Clin Cancer Res 2004; 10:4799-805; PMID:15269155; http://dx.doi.org/10.1158/1078-0432.CCR-0436-03 15269155

[16] Fritz G, Brachetti C, Bahlmann F, Schmidt M, Kaina B. Rho GTPases in human breast tumours: expression and mutation analyses and correlation with clinical parameters. Br J Cancer 2002; 87:635-44; PMID:12237774; http://dx.doi.org/10.1038/sj.bjc.6600510 12237774 PMC2364248

[17] Engers R, Ziegler S, Mueller M, Walter A, Willers R, Gabbert HE. Prognostic relevance of increased Rac GTPase expression in prostate carcinomas. Endocr Relat Cancer 2007; 14:245-56; PMID:17639041; http://dx.doi.org/10.1677/ERC-06-0036 17639041

[18] Pan Y, Bi F, Liu N, Xue Y, Yao X, Zheng Y, Fan D. Expression of seven main Rho family members in gastric carcinoma. Biochem Biophys Res Commun 2004; 315:686-91; PMID:14975755; http://dx.doi.org/10.1016/j.bbrc.2004.01.108 14975755

[19] Ji J, Feng X, Shi M, Cai Q, Yu Y, Zhu Z, Zhang J. Rac1 is correlated with aggressiveness and a potential therapeutic target for gastric cancer. Int J Oncol 2015; 46:1343-53; PMID:25585795 25585795 10.3892/ijo.2015.2836

[20] Wang JY, Yu P, Chen S, Xing H, Chen Y, Wang M, Tang K, Tian Z, Rao Q, Wang J. Activation of Rac1 GTPase promotes leukemia cell chemotherapy resistance, quiescence and niche interaction. Mol Oncol 2013; 7:907-16; PMID:23726395; http://dx.doi.org/10.1016/j.molonc.2013.05.001 23726395 PMC5528460

[21] Wang Z, Pedersen E, Basse A, Lefever T, Peyrollier K, Kapoor S, Mei Q, Karlsson R, Chrostek-Grashoff A, Brakebusch C. Rac1 is crucial for Ras-dependent skin tumor formation by controlling Pak1-Mek-Erk hyperactivation and hyperproliferation *in vivo*. Oncogene 2010; 29:3362-73; PMID:20383193; http://dx.doi.org/10.1038/onc.2010.95 20383193

[22] Heid I, Lubeseder-Martellato C, Sipos B, Mazur PK, Lesina M, Schmid RM, Siveke JT Early requirement of Rac1 in a mouse model of pancreatic cancer. Gastroenterology 2011; 141:719-30, 30 e1–7. 21684285 10.1053/j.gastro.2011.04.043

[23] Kissil JL, Walmsley MJ, Hanlon L, Haigis KM, Bender Kim CF, Sweet-Cordero A, Eckman MS, Tuveson DA, Capobianco AJ, Tybulewicz VL, et al. Requirement for Rac1 in a K-ras induced lung cancer in the mouse. Cancer Res 2007; 67:8089-94; PMID:17804720; http://dx.doi.org/10.1158/0008-5472.CAN-07-2300 17804720

[24] Myant KB, Cammareri P, McGhee EJ, Ridgway RA, Huels DJ, Cordero JB, Schwitalla S, Kalna G, Ogg EL, Athineos D, et al. ROS production and NF-kappaB activation triggered by RAC1 facilitate WNT-driven intestinal stem cell proliferation and colorectal cancer initiation. Cell Stem Cell 2013; 12:761-73; PMID:23665120; http://dx.doi.org/10.1016/j.stem.2013.04.006 23665120 PMC3690525

[25] Frances D, Sharma N, Pofahl R, Maneck M, Behrendt K, Reuter K, Krieg T, Klein CA, Haase I, Niemann C. A role for Rac1 activity in malignant progression of sebaceous skin tumors. Oncogene 2015; 34:5505-12; PMID:25659584; http://dx.doi.org/10.1038/onc.2014.471 25659584

[26] Schnelzer A, Prechtel D, Knaus U, Dehne K, Gerhard M, Graeff H, Harbeck N, Schmitt M, Lengyel E. Rac1 in human breast cancer: overexpression, mutation analysis, and characterization of a new isoform, Rac1b. Oncogene 2000; 19:3013-20; PMID:10871853; http://dx.doi.org/10.1038/sj.onc.1203621 10871853

[27] Matos P, Collard JG, Jordan P. Tumor-related alternatively spliced Rac1b is not regulated by Rho-GDP dissociation inhibitors and exhibits selective downstream signaling. J Biol Chem 2003; 278:50442-8; PMID:14506233; http://dx.doi.org/10.1074/jbc.M308215200 14506233

[28] Zhou C, Licciulli S, Avila JL, Cho M, Troutman S, Jiang P, Kossenkov AV, Showe LC, Liu Q, Vachani A, et al. The Rac1 splice form Rac1b promotes K-ras-induced lung tumorigenesis. Oncogene 2013; 32:903-9; PMID:22430205; http://dx.doi.org/10.1038/onc.2012.99 22430205 PMC3384754

[29] Stallings-Mann ML, Waldmann J, Zhang Y, Miller E, Gauthier ML, Visscher DW, Downey GP, Radisky ES, Fields AP, Radisky DC. Matrix metalloproteinase induction of Rac1b, a key effector of lung cancer progression. Sci Transl Med 2012; 4:142ra95; PMID:22786680 22786680 10.1126/scitranslmed.3004062 PMC3733503

[30] Silva AL, Carmo F, Bugalho MJ. RAC1b overexpression in papillary thyroid carcinoma: a role to unravel. Eur J Endocrinol 2013; 168:795-804; PMID:23482591; http://dx.doi.org/10.1530/EJE-12-0960 23482591

[31] Thomas EK, Cancelas JA, Zheng Y, Williams DA. Rac GTPases as key regulators of p210-BCR-ABL-dependent leukemogenesis. Leukemia 2008; 22:898-904; PMID:18354486; http://dx.doi.org/10.1038/leu.2008.71 18354486 PMC4464749

[32] Mira JP, Benard V, Groffen J, Sanders LC, Knaus UG. Endogenous, hyperactive Rac3 controls proliferation of breast cancer cells by a p21-activated kinase-dependent pathway. Proc Natl Acad Sci U S A 2000; 97:185-9; PMID:10618392; http://dx.doi.org/10.1073/pnas.97.1.185 10618392 PMC26637

[33] Faried A, Faried LS, Usman N, Kato H, Kuwano H. Clinical and prognostic significance of RhoA and RhoC gene expression in esophageal squamous cell carcinoma. Ann Surg Oncol 2007; 14:3593-601; PMID:17896152; http://dx.doi.org/10.1245/s10434-007-9562-x 17896152

[34] Mazieres J, Antonia T, Daste G, Muro-Cacho C, Berchery D, Tillement V, Pradines A, Sebti S, Favre G. Loss of RhoB expression in human lung cancer progression. Clin Cancer Res 2004; 10:2742-50; PMID:15102679; http://dx.doi.org/10.1158/1078-0432.CCR-03-0149 15102679

[35] Sato N, Fukui T, Taniguchi T, Yokoyama T, Kondo M, Nagasaka T, Goto Y, Gao W, Ueda Y, Yokoi K, et al. RhoB is frequently downregulated in non-small-cell lung cancer and resides in the 2p24 homozygous deletion region of a lung cancer cell line. Int J Cancer 2007; 120:543-51; PMID:17096327; http://dx.doi.org/10.1002/ijc.22328 17096327

[36] Arias-Romero LE, Chernoff J. Targeting Cdc42 in cancer. Expert Opin Ther Targets 2013; 17:1263-73; PMID:23957315; http://dx.doi.org/10.1517/14728222.2013.828037 23957315 PMC3937847

[37] Fritz G, Just I, Kaina B. Rho GTPases are over-expressed in human tumors. Int J Cancer 1999; 81:682-7; PMID:10328216; http://dx.doi.org/10.1002/(SICI)1097-0215(19990531)81:5%3c682::AID-IJC2%3e3.0.CO;2-B 10328216

[38] Liu Y, Wang Y, Zhang Y, Miao Y, Zhao Y, Zhang PX, Jiang GY, Zhang JY, Han Y, Lin XY, et al. Abnormal expression of p120-catenin, E-cadherin, and small GTPases is significantly associated with malignant phenotype of human lung cancer. Lung Cancer 2009; 63:375-82; PMID:19162367; http://dx.doi.org/10.1016/j.lungcan.2008.12.012 19162367

[39] Gomez Del Pulgar T, Valdes-Mora F, Bandres E, Perez-Palacios R, Espina C, Cejas P, Garcia-Cabezas MA, Nistal M, Casado E, Gonzalez-Baron M, et al. Cdc42 is highly expressed in colorectal adenocarcinoma and downregulates ID4 through an epigenetic mechanism. Int J Oncol 2008; 33:185-93; PMID:18575765 18575765

[40] Tucci MG, Lucarini G, Brancorsini D, Zizzi A, Pugnaloni A, Giacchetti A, Ricotti G, Biagini G. Involvement of E-cadherin, beta-catenin, Cdc42 and CXCR4 in the progression and prognosis of cutaneous melanoma. Br J Dermatol 2007; 157:1212-6; PMID:17970806; http://dx.doi.org/10.1111/j.1365-2133.2007.08246.x 17970806

[41] Grise F, Sena S, Bidaud-Meynard A, Baud J, Hiriart JB, Makki K, Dugot-Senant N, Staedel C, Bioulac-Sage P, Zucman-Rossi J, et al. Rnd3/RhoE Is down-regulated in hepatocellular carcinoma and controls cellular invasion. Hepatology 2012; 55:1766-75; PMID:22234932; http://dx.doi.org/10.1002/hep.25568 22234932

[42] Luo H, Dong Z, Zou J, Zeng Q, Wu D, Liu L. Down-regulation of RhoE is associated with progression and poor prognosis in hepatocellular carcinoma. J Surg Oncol 2012; 105:699-704; PMID:22213123; http://dx.doi.org/10.1002/jso.23019 22213123

[43] Zhou J, Li K, Gu Y, Feng B, Ren G, Zhang L, Wang Y, Nie Y, Fan D. Transcriptional up-regulation of RhoE by hypoxia-inducible factor (HIF)-1 promotes epithelial to mesenchymal transition of gastric cancer cells during hypoxia. Biochem Biophys Res Commun 2011; 415:348-54; PMID:22037464; http://dx.doi.org/10.1016/j.bbrc.2011.10.065 22037464

[44] Vigil D, Cherfils J, Rossman KL, Der CJ. Ras superfamily GEFs and GAPs: validated and tractable targets for cancer therapy? Nat Rev Cancer 2010; 10:842-57; PMID:21102635; http://dx.doi.org/10.1038/nrc2960 21102635 PMC3124093

[45] Barrio-Real L, Kazanietz MG. Rho GEFs and cancer: linking gene expression and metastatic dissemination. Sci Signal 2012; 5:pe43; PMID:23033535; http://dx.doi.org/10.1126/scisignal.2003543 23033535

[46] Cook DR, Rossman KL, Der CJ. Rho guanine nucleotide exchange factors: regulators of Rho GTPase activity in development and disease. Oncogene 2014; 33:4021-35; PMID:24037532; http://dx.doi.org/10.1038/onc.2013.362 24037532 PMC4875565

[47] Fields AP, Justilien V. The guanine nucleotide exchange factor (GEF) Ect2 is an oncogene in human cancer. Adv Enzyme Regul 2010; 50:190-200; PMID:19896966; http://dx.doi.org/10.1016/j.advenzreg.2009.10.010 19896966 PMC2863999

[48] Wu D, Asiedu M, Wei Q. Myosin-interacting guanine exchange factor (MyoGEF) regulates the invasion activity of MDA-MB-231 breast cancer cells through activation of RhoA and RhoC. Oncogene 2009; 28:2219-30; PMID:19421144; http://dx.doi.org/10.1038/onc.2009.96 19421144 PMC2692373

[49] Qin J, Xie Y, Wang B, Hoshino M, Wolff DW, Zhao J, Scofield MA, Dowd FJ, Lin MF, Tu Y. Upregulation of PIP3-dependent Rac exchanger 1 (P-Rex1) promotes prostate cancer metastasis. Oncogene 2009; 28:1853-63; PMID:19305425; http://dx.doi.org/10.1038/onc.2009.30 19305425 PMC2672965

[50] Berger MF, Hodis E, Heffernan TP, Deribe YL, Lawrence MS, Protopopov A, Ivanova E, Watson IR, Nickerson E, Ghosh P, et al. Melanoma genome sequencing reveals frequent PREX2 mutations. Nature 2012; 485:502-6; PMID:22622578 22622578 10.1038/nature11071 PMC3367798

[51] Habets GG, Scholtes EH, Zuydgeest D, van der Kammen RA, Stam JC, Berns A, Collard JG. Identification of an invasion-inducing gene, Tiam-1, that encodes a protein with homology to GDP-GTP exchangers for Rho-like proteins. Cell 1994; 77:537-49; PMID:7999144; http://dx.doi.org/10.1016/0092-8674(94)90216-X 7999144

[52] Adam L, Vadlamudi RK, McCrea P, Kumar R. Tiam1 overexpression potentiates heregulin-induced lymphoid enhancer factor-1/beta -catenin nuclear signaling in breast cancer cells by modulating the intercellular stability. J Biol Chem 2001; 276:28443-50; PMID:11328805; http://dx.doi.org/10.1074/jbc.M009769200 11328805

[53] Lane J, Martin TA, Mansel RE, Jiang WG. The expression and prognostic value of the guanine nucleotide exchange factors (GEFs) Trio, Vav1 and TIAM-1 in human breast cancer. Int Semin Surg Oncol 2008; 5:23; PMID:18925966; http://dx.doi.org/10.1186/1477-7800-5-23 18925966 PMC2576462

[54] Liu S, Li Y, Qi W, Zhao Y, Huang A, Sheng W, Lei B, Lin P, Zhu H, Li W, et al. Expression of Tiam1 predicts lymph node metastasis and poor survival of lung adenocarcinoma patients. Diagn Pathol 2014; 9:69; PMID:24661909; http://dx.doi.org/10.1186/1746-1596-9-69 24661909 PMC3973616

[55] Vaughan L, Tan CT, Chapman A, Nonaka D, Mack NA, Smith D, Booton R, Hurlstone AF, Malliri A. HUWE1 ubiquitylates and degrades the RAC activator TIAM1 promoting cell-cell adhesion disassembly, migration, and invasion. Cell Rep 2015; 10:88-102; PMID:25543140; http://dx.doi.org/10.1016/j.celrep.2014.12.012 25543140 PMC4542307

[56] Chen JS, Su IJ, Leu YW, Young KC, Sun HS. Expression of T-cell lymphoma invasion and metastasis 2 (TIAM2) promotes proliferation and invasion of liver cancer. Int J Cancer 2012; 130:1302-13; PMID:21469146; http://dx.doi.org/10.1002/ijc.26117 21469146

[57] Ahn SJ, Chung KW, Lee RA, Park IA, Lee SH, Park DE, Noh DY. Overexpression of betaPix-a in human breast cancer tissues. Cancer Lett 2003; 193:99-107; PMID:12691829; http://dx.doi.org/10.1016/S0304-3835(03)00004-1 12691829

[58] Fernandez-Zapico ME, Gonzalez-Paz NC, Weiss E, Savoy DN, Molina JR, Fonseca R, Smyrk TC, Chari ST, Urrutia R, Billadeau DD. Ectopic expression of VAV1 reveals an unexpected role in pancreatic cancer tumorigenesis. Cancer Cell 2005; 7:39-49; PMID:15652748; http://dx.doi.org/10.1016/j.ccr.2004.11.024 15652748

[59] Hornstein I, Pikarsky E, Groysman M, Amir G, Peylan-Ramu N, Katzav S. The haematopoietic specific signal transducer Vav1 is expressed in a subset of human neuroblastomas. J Pathol 2003; 199:526-33; PMID:12635144; http://dx.doi.org/10.1002/path.1314 12635144

[60] Lin KY, Wang LH, Hseu YC, Fang CL, Yang HL, Kumar KJ, Tai C, Uen YH. Clinical significance of increased guanine nucleotide exchange factor Vav3 expression in human gastric cancer. Mol Cancer Res 2012; 10:750-9; PMID:22544459; http://dx.doi.org/10.1158/1541-7786.MCR-11-0598-T 22544459

[61] Rao S, Lyons LS, Fahrenholtz CD, Wu F, Farooq A, Balkan W, Burnstein KL. A novel nuclear role for the Vav3 nucleotide exchange factor in androgen receptor coactivation in prostate cancer. Oncogene 2012; 31:716-27; PMID:21765461; http://dx.doi.org/10.1038/onc.2011.273 21765461 PMC3203328

[62] Citterio C, Menacho-Marquez M, Garcia-Escudero R, Larive RM, Barreiro O, Sanchez-Madrid F, Paramio JM, Bustelo XR. The rho exchange factors vav2 and vav3 control a lung metastasis-specific transcriptional program in breast cancer cells. Sci Signal 2012; 5:ra71; PMID:23033540; http://dx.doi.org/10.1126/scisignal.2002962 23033540

[63] Jarzynka MJ, Hu B, Hui KM, Bar-Joseph I, Gu W, Hirose T, Haney LB, Ravichandran KS, Nishikawa R, Cheng SY. ELMO1 and Dock180, a bipartite Rac1 guanine nucleotide exchange factor, promote human glioma cell invasion. Cancer Res 2007; 67:7203-11; PMID:17671188; http://dx.doi.org/10.1158/0008-5472.CAN-07-0473 17671188 PMC2867339

[64] Kourlas PJ, Strout MP, Becknell B, Veronese ML, Croce CM, Theil KS, Krahe R, Ruutu T, Knuutila S, Bloomfield CD, et al. Identification of a gene at 11q23 encoding a guanine nucleotide exchange factor: evidence for its fusion with MLL in acute myeloid leukemia. Proc Natl Acad Sci U S A 2000; 97:2145-50; PMID:10681437; http://dx.doi.org/10.1073/pnas.040569197 10681437 PMC15768

[65] Malliri A, van der Kammen RA, Clark K, van der Valk M, Michiels F, Collard JG. Mice deficient in the Rac activator Tiam1 are resistant to Ras-induced skin tumours. Nature 2002; 417:867-71; PMID:12075356; http://dx.doi.org/10.1038/nature00848 12075356

[66] Malliri A, Rygiel TP, van der Kammen RA, Song JY, Engers R, Hurlstone AF, Clevers H, Collard JG. The rac activator Tiam1 is a Wnt-responsive gene that modifies intestinal tumor development. J Biol Chem 2006; 281:543-8; PMID:16249175; http://dx.doi.org/10.1074/jbc.M507582200 16249175

[67] Lindsay CR, Lawn S, Campbell AD, Faller WJ, Rambow F, Mort RL, Timpson P, Li A, Cammareri P, Ridgway RA, et al. P-Rex1 is required for efficient melanoblast migration and melanoma metastasis. Nat Commun 2011; 2:555; PMID:22109529; http://dx.doi.org/10.1038/ncomms1560 22109529 PMC3400057

[68] Lissanu Deribe Y, Shi Y, Rai K, Nezi L, Amin SB, Wu CC, Akdemir KC, Mahdavi M, Peng Q, Chang QE, et al. Truncating PREX2 mutations activate its GEF activity and alter gene expression regulation in NRAS-mutant melanoma. Proc Natl Acad Sci U S A 2016; 113:E1296-305; PMID:26884185; http://dx.doi.org/10.1073/pnas.1513801113 26884185 PMC4780599

[69] Kawasaki Y, Tsuji S, Muroya K, Furukawa S, Shibata Y, Okuno M, Ohwada S, Akiyama T. The adenomatous polyposis coli-associated exchange factors Asef and Asef2 are required for adenoma formation in Apc(Min/+)mice. EMBO Rep 2009; 10:1355-62; PMID:19893577; http://dx.doi.org/10.1038/embor.2009.233 19893577 PMC2799213

[70] Chang KH, Sanchez-Aguilera A, Shen S, Sengupta A, Madhu MN, Ficker AM, Dunn SK, Kuenzi AM, Arnett JL, Santho RA, et al. Vav3 collaborates with p190-BCR-ABL in lymphoid progenitor leukemogenesis, proliferation, and survival. Blood 2012; 120:800-11; PMID:22692505; http://dx.doi.org/10.1182/blood-2011-06-361709 22692505 PMC3412345

[71] Menacho-Marquez M, Garcia-Escudero R, Ojeda V, Abad A, Delgado P, Costa C, Ruiz S, Alarcon B, Paramio JM, Bustelo XR. The Rho exchange factors Vav2 and Vav3 favor skin tumor initiation and promotion by engaging extracellular signaling loops. PLoS Biol 2013; 11:e1001615; PMID:23935450; http://dx.doi.org/10.1371/journal.pbio.1001615 23935450 PMC3720258

[72] Xue W, Krasnitz A, Lucito R, Sordella R, Vanaelst L, Cordon-Cardo C, Singer S, Kuehnel F, Wigler M, Powers S, et al. DLC1 is a chromosome 8p tumor suppressor whose loss promotes hepatocellular carcinoma. Genes Dev 2008; 22:1439-44; PMID:18519636; http://dx.doi.org/10.1101/gad.1672608 18519636 PMC2418580

[73] Ching YP, Wong CM, Chan SF, Leung TH, Ng DC, Jin DY, Ng IO. Deleted in liver cancer (DLC) 2 encodes a RhoGAP protein with growth suppressor function and is underexpressed in hepatocellular carcinoma. J Biol Chem 2003; 278:10824-30; PMID:12531887; http://dx.doi.org/10.1074/jbc.M208310200 12531887

[74] Vitiello E, Ferreira JG, Maiato H, Balda MS, Matter K. The tumour suppressor DLC2 ensures mitotic fidelity by coordinating spindle positioning and cell-cell adhesion. Nat Commun 2014; 5:5826; PMID:25518808; http://dx.doi.org/10.1038/ncomms6826 25518808 PMC4284802

[75] Wolf RM, Draghi N, Liang X, Dai C, Uhrbom L, Eklof C, Westermark B, Holland EC, Resh MD. p190RhoGAP can act to inhibit PDGF-induced gliomas in mice: a putative tumor suppressor encoded on human chromosome 19q13.3. Genes Dev 2003; 17:476-87; PMID:12600941; http://dx.doi.org/10.1101/gad.1040003 12600941 PMC196001

[76] Johnstone CN, Castellvi-Bel S, Chang LM, Bessa X, Nakagawa H, Harada H, Sung RK, Pique JM, Castells A, Rustgi AK. ARHGAP8 is a novel member of the RHOGAP family related to ARHGAP1/CDC42GAP/p50RHOGAP: mutation and expression analyses in colorectal and breast cancers. Gene 2004; 336:59-71; PMID:15225876; http://dx.doi.org/10.1016/j.gene.2004.01.025 15225876

[77] Jiang WG, Watkins G, Lane J, Cunnick GH, Douglas-Jones A, Mokbel K, Mansel RE. Prognostic value of rho GTPases and rho guanine nucleotide dissociation inhibitors in human breast cancers. Clin Cancer Res 2003; 9:6432-40; PMID:14695145 14695145

[78] Fritz G, Lang P, Just I. Tissue-specific variations in the expression and regulation of the small GTP-binding protein Rho. Biochim Biophys Acta 1994; 1222:331-8; PMID:8038201; http://dx.doi.org/10.1016/0167-4889(94)90038-8 8038201

[79] Theodorescu D, Sapinoso LM, Conaway MR, Oxford G, Hampton GM, Frierson HF Jr. Reduced expression of metastasis suppressor RhoGDI2 is associated with decreased survival for patients with bladder cancer. Clin Cancer Res 2004; 10:3800-6; PMID:15173088; http://dx.doi.org/10.1158/1078-0432.CCR-03-0653 15173088

[80] Abiatari I, DeOliveira T, Kerkadze V, Schwager C, Esposito I, Giese NA, Huber P, Bergman F, Abdollahi A, Friess H, et al. Consensus transcriptome signature of perineural invasion in pancreatic carcinoma. Mol Cancer Ther 2009; 8:1494-504; PMID:19509238; http://dx.doi.org/10.1158/1535-7163.MCT-08-0755 19509238

[81] Sahai E, Garcia-Medina R, Pouyssegur J, Vial E. Smurf1 regulates tumor cell plasticity and motility through degradation of RhoA leading to localized inhibition of contractility. J Cell Biol 2007; 176:35-42; PMID:17190792; http://dx.doi.org/10.1083/jcb.200605135 17190792 PMC2063621

[82] Castillo-Lluva S, Tan CT, Daugaard M, Sorensen PH, Malliri A. The tumour suppressor HACE1 controls cell migration by regulating Rac1 degradation. Oncogene 2013; 32:1735-42; PMID:22614015; http://dx.doi.org/10.1038/onc.2012.189 22614015

[83] Wei J, Mialki RK, Dong S, Khoo A, Mallampalli RK, Zhao Y, Zhao J. A new mechanism of RhoA ubiquitination and degradation: roles of SCF(FBXL19) E3 ligase and Erk2. Biochim Biophys Acta 2013; 1833:2757-64; PMID:23871831; http://dx.doi.org/10.1016/j.bbamcr.2013.07.005 23871831 PMC3834026

[84] Dong S, Zhao J, Wei J, Bowser RK, Khoo A, Liu Z, Luketich JD, Pennathur A, Ma H, Zhao Y. F-box protein complex FBXL19 regulates TGFbeta1-induced E-cadherin down-regulation by mediating Rac3 ubiquitination and degradation. Mol Cancer 2014; 13:76; PMID:24684802; http://dx.doi.org/10.1186/1476-4598-13-76 24684802 PMC3994216

[85] Tu S, Wu WJ, Wang J, Cerione RA. Epidermal growth factor-dependent regulation of Cdc42 is mediated by the Src tyrosine kinase. J Biol Chem 2003; 278:49293-300; PMID:14506284; http://dx.doi.org/10.1074/jbc.M307021200 14506284

[86] Preudhomme C, Roumier C, Hildebrand MP, Dallery-Prudhomme E, Lantoine D, Lai JL, Daudignon A, Adenis C, Bauters F, Fenaux P, et al. Nonrandom 4p13 rearrangements of the RhoH/TTF gene, encoding a GTP-binding protein, in non-Hodgkin's lymphoma and multiple myeloma. Oncogene 2000; 19:2023-32; PMID:10803463; http://dx.doi.org/10.1038/sj.onc.1203521 10803463

[87] Pasqualucci L, Neumeister P, Goossens T, Nanjangud G, Chaganti RS, Kuppers R, Dalla-Favera R. Hypermutation of multiple proto-oncogenes in B-cell diffuse large-cell lymphomas. Nature 2001; 412:341-6; PMID:11460166; http://dx.doi.org/10.1038/35085588 11460166

[88] Alan JK, Lundquist EA. Mutationally activated Rho GTPases in cancer. Small GTPases 2013; 4:159-63; PMID:24088985; http://dx.doi.org/10.4161/sgtp.26530 24088985 PMC3976972

[89] Cerami E, Gao J, Dogrusoz U, Gross BE, Sumer SO, Aksoy BA, Jacobsen A, Byrne CJ, Heuer ML, Larsson E, et al. The cBio cancer genomics portal: an open platform for exploring multidimensional cancer genomics data. Cancer Discov 2012; 2:401-4; PMID:22588877; http://dx.doi.org/10.1158/2159-8290.CD-12-0095 22588877 PMC3956037

[90] Gao J, Aksoy BA, Dogrusoz U, Dresdner G, Gross B, Sumer SO, Sun Y, Jacobsen A, Sinha R, Larsson E, et al. Integrative analysis of complex cancer genomics and clinical profiles using the cBioPortal. Sci Signal 2013; 6:pl1; PMID:23550210; http://dx.doi.org/10.1126/scisignal.2004088 23550210 PMC4160307

[91] Gonzalez-Perez A, Perez-Llamas C, Deu-Pons J, Tamborero D, Schroeder MP, Jene-Sanz A, Santos A, Lopez-Bigas N. IntOGen-mutations identifies cancer drivers across tumor types. Nat Methods 2013; 10:1081-2; PMID:24037244; http://dx.doi.org/10.1038/nmeth.2642 24037244 PMC5758042

[92] Hwang SL, Hong YR, Sy WD, Lieu AS, Lin CL, Lee KS, Howng SL. Rac1 gene mutations in human brain tumours. Eur J Surg Oncol 2004; 30:68-72; PMID:14736526; http://dx.doi.org/10.1016/j.ejso.2003.10.018 14736526

[93] Hodis E, Watson IR, Kryukov GV, Arold ST, Imielinski M, Theurillat JP, Nickerson E, Auclair D, Li L, Place C, et al. A landscape of driver mutations in melanoma. Cell 2012; 150:251-63; PMID:22817889; http://dx.doi.org/10.1016/j.cell.2012.06.024 22817889 PMC3600117

[94] Krauthammer M, Kong Y, Ha BH, Evans P, Bacchiocchi A, McCusker JP, Cheng E, Davis MJ, Goh G, Choi M, et al. Exome sequencing identifies recurrent somatic RAC1 mutations in melanoma. Nat Genet 2012; 44:1006-14; PMID:22842228; http://dx.doi.org/10.1038/ng.2359 22842228 PMC3432702

[95] Davis MJ, Ha BH, Holman EC, Halaban R, Schlessinger J, Boggon TJ. RAC1P29S is a spontaneously activating cancer-associated GTPase. Proc Natl Acad Sci U S A 2013; 110:912-7; PMID:23284172; http://dx.doi.org/10.1073/pnas.1220895110 23284172 PMC3549122

[96] Kawazu M, Ueno T, Kontani K, Ogita Y, Ando M, Fukumura K, Yamato A, Soda M, Takeuchi K, Miki Y, et al. Transforming mutations of RAC guanosine triphosphatases in human cancers. Proc Natl Acad Sci U S A 2013; 110:3029-34; PMID:23382236; http://dx.doi.org/10.1073/pnas.1216141110 23382236 PMC3581941

[97] Watson IR, Li L, Cabeceiras PK, Mahdavi M, Gutschner T, Genovese G, Wang G, Fang Z, Tepper JM, Stemke-Hale K, et al. The RAC1 P29S hotspot mutation in melanoma confers resistance to pharmacological inhibition of RAF. Cancer Res 2014; 74:4845-52; PMID:25056119; http://dx.doi.org/10.1158/0008-5472.CAN-14-1232-T 25056119 PMC4167745

[98] Van Allen EM, Wagle N, Sucker A, Treacy DJ, Johannessen CM, Goetz EM, Place CS, Taylor-Weiner A, Whittaker S, Kryukov GV, et al. The genetic landscape of clinical resistance to RAF inhibition in metastatic melanoma. Cancer Discov 2014; 4:94-109; PMID:24265153; http://dx.doi.org/10.1158/2159-8290.CD-13-0617 24265153 PMC3947264

[99] Mar VJ, Wong SQ, Logan A, Nguyen T, Cebon J, Kelly JW, Wolfe R, Dobrovic A, McLean C, McArthur GA. Clinical and pathological associations of the activating RAC1 P29S mutation in primary cutaneous melanoma. Pigment Cell Melanoma Res 2014; 27:1117-25; PMID:25043693; http://dx.doi.org/10.1111/pcmr.12295 25043693

[100] Vu HL, Rosenbaum S, Purwin TJ, Davies MA, Aplin AE. RAC1 P29S regulates PD-L1 expression in melanoma. Pigment Cell Melanoma Res 2015; 28:590-8; PMID:26176707; http://dx.doi.org/10.1111/pcmr.12392 26176707 PMC4675336

[101] Thomas EK, Cancelas JA, Chae HD, Cox AD, Keller PJ, Perrotti D, Neviani P, Druker BJ, Setchell KD, Zheng Y, et al. Rac guanosine triphosphatases represent integrating molecular therapeutic targets for BCR-ABL-induced myeloproliferative disease. Cancer Cell 2007; 12:467-78; PMID:17996650; http://dx.doi.org/10.1016/j.ccr.2007.10.015 17996650

[102] Cho YJ, Zhang B, Kaartinen V, Haataja L, de Curtis I, Groffen J, Heisterkamp N. Generation of rac3 null mutant mice: role of Rac3 in Bcr/Abl-caused lymphoblastic leukemia. Mol Cell Biol 2005; 25:5777-85; PMID:15964830; http://dx.doi.org/10.1128/MCB.25.13.5777-5785.2005 15964830 PMC1157002

[103] Cancer Genome Atlas Research N. Comprehensive molecular characterization of gastric adenocarcinoma. Nature 2014; 513:202-9; PMID:25079317; http://dx.doi.org/10.1038/nature13480 25079317 PMC4170219

[104] Kakiuchi M, Nishizawa T, Ueda H, Gotoh K, Tanaka A, Hayashi A, Yamamoto S, Tatsuno K, Katoh H, Watanabe Y, et al. Recurrent gain-of-function mutations of RHOA in diffuse-type gastric carcinoma. Nat Genet 2014; 46:583-7; PMID:24816255; http://dx.doi.org/10.1038/ng.2984 24816255

[105] Palomero T, Couronne L, Khiabanian H, Kim MY, Ambesi-Impiombato A, Perez-Garcia A, Carpenter Z, Abate F, Allegretta M, Haydu JE, et al. Recurrent mutations in epigenetic regulators, RHOA and FYN kinase in peripheral T cell lymphomas. Nat Genet 2014; 46:166-70; PMID:24413734; http://dx.doi.org/10.1038/ng.2873 24413734 PMC3963408

[106] Yoo HY, Sung MK, Lee SH, Kim S, Lee H, Park S, Kim SC, Lee B, Rho K, Lee JE, et al. A recurrent inactivating mutation in RHOA GTPase in angioimmunoblastic T cell lymphoma. Nat Genet 2014; 46:371-5; PMID:24584070; http://dx.doi.org/10.1038/ng.2916 24584070

[107] Manso R, Sanchez-Beato M, Monsalvo S, Gomez S, Cereceda L, Llamas P, Rojo F, Mollejo M, Menarguez J, Alves J, et al. The RHOA G17V gene mutation occurs frequently in peripheral T-cell lymphoma and is associated with a characteristic molecular signature. Blood 2014; 123:2893-4; PMID:24786457; http://dx.doi.org/10.1182/blood-2014-02-555946 24786457

[108] Rodrigues P, Macaya I, Bazzocco S, Mazzolini R, Andretta E, Dopeso H, Mateo-Lozano S, Bilic J, Carton-Garcia F, Nieto R, et al. RHOA inactivation enhances Wnt signalling and promotes colorectal cancer. Nat Commun 2014; 5:5458; PMID:25413277; http://dx.doi.org/10.1038/ncomms6458 25413277 PMC4255233

[109] Richter J, Schlesner M, Hoffmann S, Kreuz M, Leich E, Burkhardt B, Rosolowski M, Ammerpohl O, Wagener R, Bernhart SH, et al. Recurrent mutation of the ID3 gene in Burkitt lymphoma identified by integrated genome, exome and transcriptome sequencing. Nat Genet 2012; 44:1316-20; PMID:23143595; http://dx.doi.org/10.1038/ng.2469 23143595

[110] Rohde M, Richter J, Schlesner M, Betts MJ, Claviez A, Bonn BR, Zimmermann M, Damm-Welk C, Russell RB, Borkhardt A, et al. Recurrent RHOA mutations in pediatric Burkitt lymphoma treated according to the NHL-BFM protocols. Genes Chromosomes Cancer 2014; 53:911-6; PMID:25044415; http://dx.doi.org/10.1002/gcc.22202 25044415

[111] Sahai E, Alberts AS, Treisman R. RhoA effector mutants reveal distinct effector pathways for cytoskeletal reorganization, SRF activation and transformation. EMBO J 1998; 17:1350-61; PMID:9482732; http://dx.doi.org/10.1093/emboj/17.5.1350 9482732 PMC1170483

[112] Cleverley SC, Costello PS, Henning SW, Cantrell DA. Loss of Rho function in the thymus is accompanied by the development of thymic lymphoma. Oncogene 2000; 19:13-20; PMID:10644975; http://dx.doi.org/10.1038/sj.onc.1203259 10644975

[113] Cancer Genome Atlas Research N. Comprehensive molecular characterization of urothelial bladder carcinoma. Nature 2014; 507:315-22; PMID:24476821; http://dx.doi.org/10.1038/nature12965 24476821 PMC3962515

[114] Liu AX, Rane N, Liu JP, Prendergast GC. RhoB is dispensable for mouse development, but it modifies susceptibility to tumor formation as well as cell adhesion and growth factor signaling in transformed cells. Mol Cell Biol 2001; 21:6906-12; PMID:11564874; http://dx.doi.org/10.1128/MCB.21.20.6906-6912.2001 11564874 PMC99867

[115] Hakem A, Sanchez-Sweatman O, You-Ten A, Duncan G, Wakeham A, Khokha R, Mak TW. RhoC is dispensable for embryogenesis and tumor initiation but essential for metastasis. Genes Dev 2005; 19:1974-9; PMID:16107613; http://dx.doi.org/10.1101/gad.1310805 16107613 PMC1199568

[116] van Hengel J, D'Hooge P, Hooghe B, Wu X, Libbrecht L, De Vos R, Quondamatteo F, Klempt M, Brakebusch C, van Roy F. Continuous cell injury promotes hepatic tumorigenesis in cdc42-deficient mouse liver. Gastroenterology 2008; 134:781-92; PMID:18325391; http://dx.doi.org/10.1053/j.gastro.2008.01.002 18325391

[117] Gao Y, Dickerson JB, Guo F, Zheng J, Zheng Y. Rational design and characterization of a Rac GTPase-specific small molecule inhibitor. Proc Natl Acad Sci U S A 2004; 101:7618-23; PMID:15128949; http://dx.doi.org/10.1073/pnas.0307512101 15128949 PMC419655

[118] Onesto C, Shutes A, Picard V, Schweighoffer F, Der CJ. Characterization of EHT 1864, a novel small molecule inhibitor of Rac family small GTPases. Methods Enzymol 2008; 439:111-29; PMID:18374160; http://dx.doi.org/10.1016/S0076-6879(07)00409-0 18374160

[119] Kaneto N, Yokoyama S, Hayakawa Y, Kato S, Sakurai H, Saiki I. RAC1 inhibition as a therapeutic target for gefitinib-resistant non-small-cell lung cancer. Cancer Sci 2014; 105:788-94; PMID:24750242; http://dx.doi.org/10.1111/cas.12425 24750242 PMC4317907

[120] Dutting S, Heidenreich J, Cherpokova D, Amin E, Zhang SC, Ahmadian MR, Brakebusch C, Nieswandt B. Critical off-target effects of the widely used Rac1 inhibitors NSC23766 and EHT1864 in mouse platelets. J Thromb Haemost 2015; 13:827-38; PMID:25628054; http://dx.doi.org/10.1111/jth.12861 25628054

[121] Cardama GA, Comin MJ, Hornos L, Gonzalez N, Defelipe L, Turjanski AG, Alonso DF, Gomez DE, Menna PL. Preclinical development of novel Rac1-GEF signaling inhibitors using a rational design approach in highly aggressive breast cancer cell lines. Anticancer Agents Med Chem 2014; 14:840-51; PMID:24066799; http://dx.doi.org/10.2174/18715206113136660334 24066799 PMC4104455

[122] Fine B, Hodakoski C, Koujak S, Su T, Saal LH, Maurer M, Hopkins B, Keniry M, Sulis ML, Mense S, et al. Activation of the PI3K pathway in cancer through inhibition of PTEN by exchange factor P-REX2a. Science 2009; 325:1261-5; PMID:19729658; http://dx.doi.org/10.1126/science.1173569 19729658 PMC2936784

[123] Guo Y, Kenney SR, Cook L, Adams SF, Rutledge T, Romero E, Oprea TI, Sklar LA, Bedrick E, Wiggins CL, et al. A Novel Pharmacologic Activity of Ketorolac for Therapeutic Benefit in Ovarian Cancer Patients. Clin Cancer Res 2015; 21:5064-72; PMID:26071482; http://dx.doi.org/10.1158/1078-0432.CCR-15-0461 26071482 PMC4644688

[124] Guo Y, Kenney SR, Muller CY, Adams S, Rutledge T, Romero E, Murray-Krezan C, Prekeris R, Sklar LA, Hudson LG, et al. R-Ketorolac Targets Cdc42 and Rac1 and Alters Ovarian Cancer Cell Behaviors Critical for Invasion and Metastasis. Mol Cancer Ther 2015; 14:2215-27; PMID:26206334; http://dx.doi.org/10.1158/1535-7163.MCT-15-0419 26206334 PMC4596774

[125] Zins K, Lucas T, Reichl P, Abraham D, Aharinejad S. A Rac1/Cdc42 GTPase-specific small molecule inhibitor suppresses growth of primary human prostate cancer xenografts and prolongs survival in mice. PLoS One 2013; 8:e74924; PMID:24040362; http://dx.doi.org/10.1371/journal.pone.0074924 24040362 PMC3770583

[126] Pelish HE, Peterson JR, Salvarezza SB, Rodriguez-Boulan E, Chen JL, Stamnes M, Macia E, Feng Y, Shair MD, Kirchhausen T. Secramine inhibits Cdc42-dependent functions in cells and Cdc42 activation *in vitro*. Nat Chem Biol 2006; 2:39-46; PMID:16408091; http://dx.doi.org/10.1038/nchembio751 16408091

[127] Friesland A, Zhao Y, Chen YH, Wang L, Zhou H, Lu Q. Small molecule targeting Cdc42-intersectin interaction disrupts Golgi organization and suppresses cell motility. Proc Natl Acad Sci U S A 2013; 110:1261-6; PMID:23284167; http://dx.doi.org/10.1073/pnas.1116051110 23284167 PMC3557054

[128] Zins K, Gunawardhana S, Lucas T, Abraham D, Aharinejad S. Targeting Cdc42 with the small molecule drug AZA197 suppresses primary colon cancer growth and prolongs survival in a preclinical mouse xenograft model by downregulation of PAK1 activity. J Transl Med 2013; 11:295; PMID:24279335; http://dx.doi.org/10.1186/1479-5876-11-295 24279335 PMC4222769

[129] Rath N, Olson MF. Rho-associated kinases in tumorigenesis: re-considering ROCK inhibition for cancer therapy. EMBO Rep 2012; 13:900-8; PMID:22964758; http://dx.doi.org/10.1038/embor.2012.127 22964758 PMC3463970

[130] Somlyo AV, Bradshaw D, Ramos S, Murphy C, Myers CE, Somlyo AP. Rho-kinase inhibitor retards migration and *in vivo* dissemination of human prostate cancer cells. Biochem Biophys Res Commun 2000; 269:652-9; PMID:10720471; http://dx.doi.org/10.1006/bbrc.2000.2343 10720471

[131] Itoh K, Yoshioka K, Akedo H, Uehata M, Ishizaki T, Narumiya S. An essential part for Rho-associated kinase in the transcellular invasion of tumor cells. Nat Med 1999; 5:221-5; PMID:9930872; http://dx.doi.org/10.1038/5587 9930872

[132] Kumar MS, Hancock DC, Molina-Arcas M, Steckel M, East P, Diefenbacher M, Armenteros-Monterroso E, Lassailly F, Matthews N, Nye E, et al. The GATA2 transcriptional network is requisite for RAS oncogene-driven non-small cell lung cancer. Cell 2012; 149:642-55; PMID:22541434; http://dx.doi.org/10.1016/j.cell.2012.02.059 22541434

